# The Preston Retreat

**Published:** 1889-08

**Authors:** 


THE PRESTON RETREAT.—Dr. A. Lapthorn Smith, of Montreal, who lately paid a visit to Philadelphia, writes a most pleasant letter to the *Canada Medical Record*, published in the July number, in which he thus describes this model maternity:

"On my arrival I proceeded at once to the 'Preston Retreat,' at 500 North Twentieth street, and reported myself to Dr. Joseph Price, the Medical Director. Perhaps I had better explain what the 'Preston Retreat' is. An old Dr. Preston, many years ago, left a large amount of money in the hands of trustees for the purpose of building and endowing a model maternity institution. The funds at their disposal were so considerable as to place absolutely no limit on any expenditure which might be necessary in any way towards reducing the death-rate of midwifery cases; so that as science suggested improvements from time to time, the trustees have carried them out. The position of this institution is peculiar. First of all, only married women are eligible for admission. Second, it has a paid resident physician; not paid in the ordinary sense of the term, such as two or three hundred dollars a year and board, which would be thought a large sum in Canada or England for such an officer, but paid to the extent of some five thousand dollars a year, with a magnificent private residence free. For these inducements they can get one of the best men. When I say that Goodell held this position for twenty years, only resigning it two years ago, when Dr. Joseph Price was appointed, you will admit that nothing is spared in that direction. And when I add that each confinement costs the nice little sum of two hundred dollars, including a six week's stay in the Retreat, you will see that it has everything in its favor to make it a model establishment. It has one other peculiarity which contributes enormously to its success, namely, it has no students coming in from the dissecting-room and surgical wards to carry death on their fingers. A series of 500 confinements has just been published without a death, although among them were fifty-two cases of instrumental delivery, many of the mothers having contracted pelves. There was one case of placenta prævia, three of twins, also several face and breech presentations. The secrets of success seem to be absolute cleanliness of persons and surroundings, and abundance of water, soap, and pure air. As a rule, but one digital examination is made. As the head passes through the vulva, the attendant washes the child's eyes with a piece of jute moistened in sublimate solution, so that there has only been one case of ophthalmia neonatorum in 500 births. Immediately after the delivery, the vagina is washed out with clean boiled water, injuries to the vulva are at once repaired, the clothes are changed, an antiseptic pad applied to the vulva, and the patient is put to bed in the ward. As soon as a ward has received its tenth patient, another ward is opened up, and when it is full, another. In the meantime, the patients are moved out of the first ward at the end of ten days, so that in twenty days from its opening all the patients will have passed on to the convalescent ward, while the first ward is thoroughly cleaned out and left to air until its turn for occupation comes around again. Every two hours a laundry girl makes the rounds of the hospital with a closed basket, and gathers up all soiled linen and takes it off to the laundry, which is situated in a separate building. There are no water-closets in the house, but at the four corners of the main building there are detached towers, connected with it by galleries closed in with glass in Winter but open in Summer, and in these towers are placed all the baths and water-closets. The wards are so placed as to have three sides exposed to the air and sunshine. The mattresses are filled with straw, which is put fresh into a clean tick for each patient. Instead of napkins, antiseptic pads are used to absorb the lochia. They are made as follows: a napkin of soft loose-textured cotton is laid on the table; on it is placed a sheet of waxed paper, which any one can make; then a handful of sublimated jute is laid in the center, then a layer of absorbent cotton, and finally the napkin is caught up at the sides with a few threads. Several hundred of these are kept in stock, and, of course, they are burned when soiled. It is not often that we are able to carry out our ideal of what things should be, but in the case of the Preston Retreat there is nothing to prevent it from being a model maternity, and it is one. Every mother must nurse her child, which is put to the breast as soon as it is washed, and Dr. Price tells me he has never seen a suppurating breast.

"It may be noticed that the ratio of forceps cases is very moderate, about one in ten, which is probably another secret of success. The temptation to use them must be very great, for the attendant is allowed to engage in private practice, and is one of the busiest men in Philadelphia. On the evening of my arrival, no less than three practitioners called in to engage his services in cases of laparatomy, for it is in this branch of gynecology that he is best known. He is an ardent follower of Tait, believing that abdominal section is the best, quickest, and safest treatment for nearly all diseases of the female pelvic organs. Thus, ovarian cysts, fibroid tumors, malignant disease, adherent ovaries especially if prolapsed, enlarged tubes especially if adherent, pyo-, hydro-, and hemato-salpinx, extrauterine fetation should all be treated by removal alone. Especially does he abhor electricity in every shape and form. He is a young man, probably less than thirty-five, quick in speech and action, with deep-set eyes, which give him an intensely earnest expression. He began his career in the out-patient department of the Pennsylvania Hospital, after having been a pupil of Goodell. He first came into notice by reason of his success in abdominal sections performed at the domiciles of the poor, often in the filthiest courts and streets in the city, his results being better than is usually obtained in the best appointed hospitals. He was enabled to do this by organizing a voluntary nurses' association, composed of young ladies who would go to a rickety house the day before an operation and make the patient and her rooms clean, the former with soap and water and the latter with whitewash. This association also supplied a clean bed, bedding, and night clothes. Others took charge of the patient on the day of the operation. Instead of chemical disinfectants, he uses distilled water, with which he freely floods out the abdominal cavity. The day after my arrival he took me to see some of his cases. One of them, a case of vaginal hysterectomy performed at a private boarding-house, was in charge of a nurse, a bright young girl of nineteen or twenty, whom he asked to show me her watch. He had promised to give her a gold watch if she succeeded in nursing forty-five cases of abdominal section in which a drainage-tube had been inserted without a death. These were all cases in which there had been serious adhesions and a good deal of oozing, which this faithful girl had removed every half-hour with a syringe until the tubes were no longer required. The fact was duly inscribed on her watch, of which she was justly proud. Dr. Price tells me that he will have no nurse who was trained before he got her. He wants an intelligent, fairly educated young girl, without any professional knowledge, whom he puts to work at once under the direction of a more experienced one, whom she relieves at stated intervals. I should say, however, that he presents each with two or three good books on nursing. He never attempts an operation without one or two of these young girls to take the case in hand afterwards. As he performs an operation two or three times a week, he must have a number of them on hand. He sends them out to the mining towns around Philadelphia, where, in the miners' cottages, they have often to make their bed on two or three chairs, but they never murmur. It is a pleasure to see him operate, for two or three reasons. One is the smallness of the abdominal incision, which is barely large enough to admit two fingers of the left hand. The intestines are never seen. Another pleasure is the rapidity with which he operates, between six and ten minutes being the average. And the third noticeable feature is the fewness of his tools; the same little scalpel which has done over two hundred sections, three Pean's forceps, one blunt Peaslee's needle armed with a boiled silk ligature for the pedicle, and a triangular needle with the same for the abdominal sutures. I was almost forgetting what, in his estimation, is one of the most important of all, an enameled iron funnel, with a good-sized tube and a perforated silver-plated round-ended tube with which the cavity is washed out with boiled or distilled water. This irrigator is introduced to the very bottom of Douglas's pouch. Absolutely nothing is given during the first twenty-four hours, no opium, not even a drop of ice-water. If the patient has not passed flatus at the end of that time, small doses of Rochelle salts are given until she does. It may be asked, Is there not too much of this abdominal section? Assuredly there is. But I must say this, I did not see one case operated on in which there was not grossly evident disease of the tubes or ovaries, or else a firm binding down together of these organs by localized peritonitis. Dr. Price insists upon visitors remaining after the operation long enough to see the specimen floated in water, when the long shreds of torn adhesions become strikingly evident. He is a firm believer in gonorrheal infection of the tubes and peritoneum, and where this could not be, then a "dirty" confinement is blamed for these cases."

